# Optimization of row-ratios in mechanized hybrid rice seed production: a study on pollen dispersal and seed-setting characteristics at two ecological sites

**DOI:** 10.3389/fpls.2025.1704773

**Published:** 2025-12-16

**Authors:** Ziyu Li, Guangyi Chen, Wei Li, Hong Yang, Pengwang Guo, Conghua Zhu, Li Zhang, Yao Zhang, Junqi Yu, Xi Luo, Tian Li, Yuyuan Ouyang, Xuyi Li

**Affiliations:** 1 Crop Research Institute, Sichuan Academy of Agricultural Sciences, Chengdu, China; 2 College of Agronomy, Sichuan Agricultural University, Chengdu, China; 3 College of Agronomy, South China Agricultural University, Guangzhou, China; 4 Rice Research Institute, Sichuan Agricultural University, Chengdu, China; 5 Sichuan Provincial Key Laboratory of Green Germplasm Innovation and Genetic Improvement for Grain and Oil Crops, Chengdu, China

**Keywords:** hybrid rice, mechanized seed production, row-ratio, pollen dispersal, yield

## Abstract

To explore the optimal row-ratio in mechanized hybrid rice seed production, a field experiment was conducted in 2024 at Qionglai and Mianzhu using ‘Tiantai A’ × ‘Taihui 808’. Three row-ratio treatments (H1: 18:6, H2: 24:6, and H3: 30:6) were tested using agricultural unmanned aerial vehicles (AUAVs) for pollination assistance. The results showed that row-ratio had little effect on sterile line flowering dynamics. The index of flowers meeting (IFM) was 0.71-0.72 at Qionglai and 0.81–0.86 at Mianzhu, with 11 to 12 days of flowering duration. As the row-ratio increased, total pollen quantity in the panicle layer and grain filling rate (GFR) decreased, while grain infection rate (GIR) increased. The responses of grain blighted rate (GBR), grain empty rate (GER), and fertilization success rate (FSR) to row-ratio varied between sites. Pollen density and GFR followed the pattern of near region (NR) > central region (CR) > far region (FR). Within the panicle, pollen density was generally highest in the upper panicle layer (UPL), followed by the middle (MPL) and lower (LPL) layers, with partial exceptions observed in the H2 and H3 treatments at Mianzhu. The vertical distribution of GFR varied by site: at Qionglai, it was apical parts of panicle (APP) > median parts (MPP) > basal parts (BPP), whereas at Mianzhu the order was MPP > APP > BPP. With wider row-ratios, yield per unit area (YUA) and GFR declined (H1 > H2 > H3), while 1,000-grain weight increased or decreased and then increased. Under H1, yields reached 2,107.50 kg ha^−1^ at Qionglai and 2,201.62 kg ha^−1^ at Mianzhu, 4.18% and 3.18% higher than H2 and 6.74% and 5.77% higher than H3. However, the equivalent monoculture yield per hectare (EMYH) increased with wider row-ratios (H3 > H2 > H1). Under H3, equivalent yields were 1,645.43 kg ha^−1^ at Qionglai and 1,734.59 kg ha^−1^ at Mianzhu, increasing by 4.10% and 1.68% over H1 and 5.05% and 1.62% over H2. In summary, although wider row-ratios reduced pollen supply and GFR per sterile row, the increased number of sterile rows compensated, enhancing total seed production. Thus, the 30:6 row-ratio (H3) is recommended for optimal yield.

## 1 Introduction

Seeds are regarded as the “chips” of agriculture, with seed production serving as a key industry to ensure food security and a central driver of agricultural modernization (Luo, 2023; Xu et al., 2022). Within the hybrid rice production system, the seed production stage is the foundational process, and its technological level directly determines the seed yield, quality, and production costs. At present, a pronounced mismatch exists between agronomic practices and mechanization in hybrid rice seed production. The unique field layout required for seed production further exacerbates the incompatibility with mechanized operations, manifesting mainly in poor compatibility with existing mechanization systems and low efficiency of manual pollination (Huang, 2015). These constraints not only limit the economic benefits of hybrid rice seed production but also undermine seed supply security and quality stability, making it difficult to meet the emerging demands of modern seed industry development (Chen et al., 2015; Kong et al., 2023). Against this backdrop, the deep integration of mechanization and agronomic practices offers a promising pathway to leverage the advantages of mechanized operations in large-scale seed production, thereby ensuring efficiency and stability in hybrid rice seed production (Ding et al., 2021; Tao et al., 2022).

Recent studies have identified a critical challenge in mechanized hybrid rice seed production: the incompatibility between the traditional small row-ratio patterns used in manual seed production (e.g., 6–10:2) and mechanized farming systems. These conventional designs were developed for labor-intensive operations and are poorly aligned with the fixed working widths of transplanting and harvesting machinery. Currently, seed production fields mainly rely on general-purpose rice machinery. Transplanting is typically carried out with 6–8-row transplanters (e.g., Kubota NSPU-68C, Yanmar YR60DDA), while harvesting predominantly employs full-feed combine harvesters (e.g., Lovol Gushen RG50, World Haolong 4LZ-5.0E) with cutting widths of 2.0–2.5 m. The narrow block width of the restorer lines, which is less than the cutting width of standard harvesters, and the lack of dedicated machinery make it impossible to harvest these rows separately to prevent admixture. Compounded by the immaturity and limited adoption of seed sorting technology, these factors present a major challenge to harvesting operations (Tang et al., 2020). In addition, traditional manual pollination methods (e.g., the single-pole method, the double-pole method, or rope pulling) are highly labor-intensive and fail to ensure sufficient pollen distribution uniformity and coverage. In recent years, advances in agricultural unmanned aerial vehicles (AUAV) pollination technology have provided new opportunities to expand row-ratios and enhance pollen dispersal efficiency (Jiang et al., 2025). However, these advances have also highlighted the urgent need to reform the small row-ratio model to achieve full mechanization, particularly by addressing the mismatch between restorer block width and harvester cutting width. Adjustments to row-ratios, however, may result in excessive pollen supply with increasing numbers of restorer rows while simultaneously reducing the planting area available for sterile rows. This necessitates a proportional increase in sterile rows, leading to wider parental row-ratios. Despite its potential importance, the applicability of large row-ratios to “three-line” hybrid rice systems remains poorly understood. Furthermore, the interactive effects of increased parental row numbers on land-use efficiency and pollen dispersal attenuation have not been systematically investigated (Lu, 2023; Zhang, 2022).

Previous studies have demonstrated that the configuration of parental row-ratios determines the proportional distribution of sterile lines and restorer lines per unit area, thereby influencing the uniformity of pollen density and the rationality of population structure in seed production fields (Huang et al., 2022). The spatial distribution of restorer pollen directly dictates the seed-setting of the sterile line, with rows closer to the restorer line receiving significantly more pollen and achieving a higher seed setting rate than those farther away (Sun et al., 2020). It is reported that moderately widening the row-ratio allows AUAVs to generate a sufficiently broad wind field to achieve full pollination coverage of the sterile line (Li et al., 2015). In contrast, some studies emphasized that excessively wide row-ratios may hinder effective pollen transport to distant sterile rows, even with AUAV assistance, thereby reducing pollination efficiency (He et al., 2022). Further evidence showed that expanding the row-ratio (8–26:2) increased the number of effective panicles per unit area in the sterile line but decreased the seed setting rate (Xu et al., 2007). Within a certain range, the increase in panicle number could compensate for the decline in seed setting rate and maintain yield, whereas beyond this threshold, yield declined.

Therefore, optimizing parental row-ratios—balancing the sterile line population size (panicle number) with effective pollen coverage (seed setting rate)—is critical to maximize yield. However, most previous studies have focused on small row-ratios under manual pollination, with limited attention to the regulatory role of field configuration parameters in mechanized seed production. Given the rapid development of mechanized hybrid rice seed production, it is urgent to establish field configuration models compatible with mechanized transplanting and harvesting while improving pollen capture efficiency of the sterile line to meet the requirements of full mechanization. To address this need, the present study established three large parental row-ratio treatments suitable for mechanized sowing, transplanting, and harvesting and systematically analyzed their effects on pollination efficiency and seed-setting characteristics. The findings aim to provide theoretical and technical guidance for high-yield cultivation in mechanized hybrid rice seed production.

## 2 Materials and methods

### 2.1 Experimental site and materials

This experiment was conducted from April to September 2024 at two ecological sites, Qionglai (30°24′ N, 103°31′ E) and Mianzhu (31°15′ N, 104°13′ E) in Sichuan Province, China. Prior to the field trials, soil samples from the topsoil layer (0–20 cm) were analyzed. The basic physicochemical properties of the soil are presented in **Supplementary Table S1**, while essential meteorological data are shown in **Supplementary Figure S1**. The sterile line used was indica rice ‘Tiantai A’, and the restorer line was indica conventional rice ‘Taihui 808’ (growth periods of approximately 100 and 125 days, respectively). These materials were provided by Sichuan Academy of Agricultural Sciences and bred by Sichuan Tailong Huizhi Biotechnology Co., Ltd.

### 2.2 Experimental design

A randomized complete block design with three replicates was implemented across a total experimental area of 930 m². Three row-ratio treatments of sterile line to restorer line were established: 18:6 (H1), 24:6 (H2), and 30:6 (H3). The row spacing × plant spacing was set at 30 cm × 16 cm for sterile lines and 30 cm × 20 cm for restorer lines, with a 30-cm inter-row gap between parental blocks. Mechanical transplanting employed the blanket seedling technique. At Qionglai, the restorer line was sown on April 11 and transplanted on May 13, while the sterile line was sown on May 5 and transplanted on May 30. At Mianzhu, the restorer line was sown on April 12 and transplanted on May 14, while the sterile line was sown on May 6 and transplanted on May 31.

Throughout the parental growth period, a total pure nitrogen application of 180 kg ha^−1^ was administered. The total basal fertilizer comprised 600 kg ha^−1^ compound fertilizer (N/P_2_O_5_/K_2_O = 15:15:15) supplemented with 78.26 kg ha^−1^ urea (N, 46.4%). At 5 days after transplanting, 117.39 kg ha^−1^ urea was applied to both restorer and sterile lines. For the sterile line exclusively, potassium chloride (K_2_O, 60.0%) was applied at 300 kg ha^−1^, distributed in a 1:1 ratio as basal fertilizer to panicle-promoting fertilizer.

Hexacopter-configured AUAVs (**Supplementary Table S2**) were deployed for supplementary pollination. During flowering, two to three pollen dispersal operations were conducted daily between 11:00 and 13:00 h. AUAVs operated at flight altitudes of 1.5-2.0 m above the restorer line panicle layer, with velocities maintained at 4 to 5 m s^−1^ under moderate wind conditions, traversing parallel to restorer rows. Transplanting and harvesting operations were carried out using a Yanmar YR60D (six-row) rice transplanter and a Kubota PRO688Q combine harvester (2.5-m cutting platform), respectively. Field management, including the prevention and control of pests and weeds, was conducted according to local cultural practices.

### 2.3 Measurements and methods

#### 2.3.1 Flowering dynamics of parental lines

At the initial heading stage, three hills with similar growth progress were selected based on the average number of tillers per hill. Starting from the emergence of the first panicle, after 16:00 daily, the number of flowers opening on each panicle per plant was recorded based on the presence or absence of anthers or their arrangement (neat or scattered). Flowers were removed after recording. This continued until flowering completion per plant, after which the index of flowers meeting (IFM) was calculated.


$$IFM=\sum (f_{i}\times M+m_{i}\times F-|f_{i}\times M–m_{i}\times F|)/\left(2\times F\times M\right)\;(i=1,2,3,\ldots .n)$$

where “i” is the number of days from the first flowering day to the end of the flowering period in the sterile line; “fi” and “mi” represent the daily flowering counts of the sterile line and restorer line, respectively, on day “i” of the sterile line’s flowering period; and “FM” denotes the product of the total flowering counts of the restorer line and sterile line.

#### 2.3.2 Pollen distribution in sterile line regions and panicle layers

**Figure 1:**
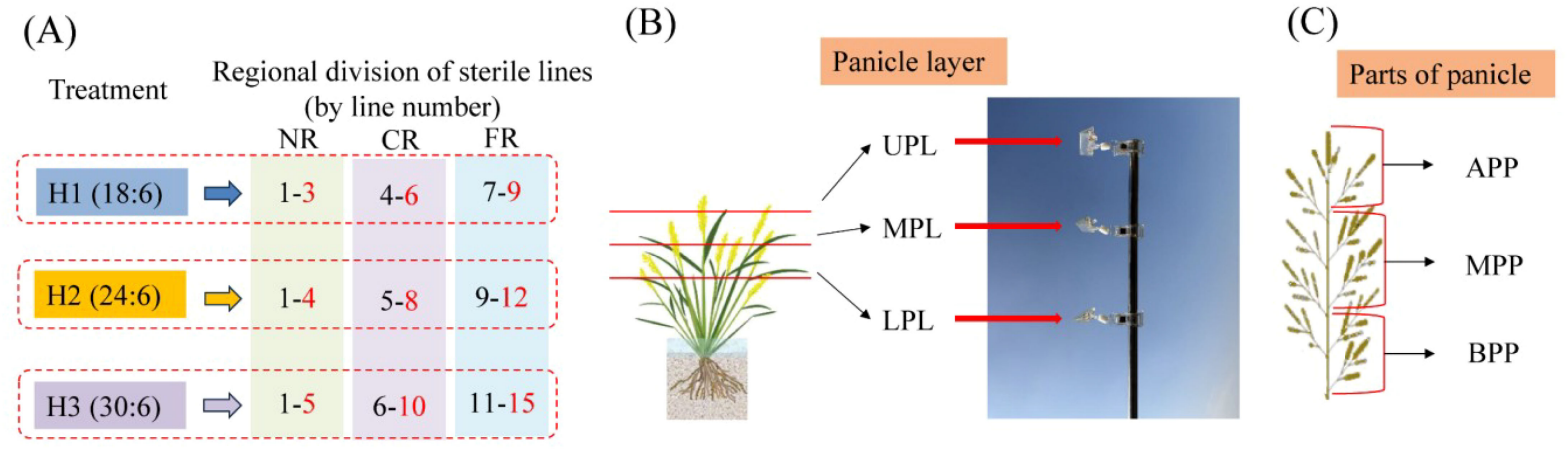
Definition of sterile line regions, panicle layers, parts of panicle, and fixed points for pollen collection in the field. Sampling points were positioned in the farthest rows within each region. For H1, rows 3, 6, and 9 were NR, CR, and FR points, respectively. For H2, rows 4, 8, and 12 served as NR, CR, and FR points, respectively. For H3, rows 5, 10, and 15 represented NR, CR, and FR points, respectively. ULP, MLP, LPL, APP, MPP, and BPP represent upper panicle layer, middle panicle layer, lower panicle layer, apical parts of panicle, median parts of panicle, and basal parts of panicle, respectively.

Pollen quantity in different rows was measured using the aerial pollen capture method. Observation points for the sterile line were set on the same side as the restorer line in each treatment, specifically at the farthest row within the near region (NR), central region (CR), and far region (FR) of the sterile line (**Figure 1A**). At each observation point, glass slides were placed at equal heights in the upper panicle layer (UPL), middle panicle layer (MPL), and lower panicle layer (LPL) of the sterile line’s panicle canopy (**Figure 1B**) to collect pollen. Before pollination, polyvinyl chloride pipes were inserted at panicle layer height, and glass slides coated with Vaseline (coated surface facing upward) were fixed on the pipes using clips. After pollination, the glass slides were retrieved, stained with a 2% I_2_–KI solution, and observed under a microscope (×10 eyepiece, ×10 objective; field of view area approximately 1219.2 μm × 914.4 μm). The number of normally stained pollen grains in five fields of view was counted, and the average number represented the pollen quantity at the observation point.

#### 2.3.3 Grain types of sterile lines

At maturation, 60 hills were selected to investigate the average number of tillers. Then, three hills of representative plants were selected from each region (NR, CR, and FR) per treatment, with three replicates. The number of primary branches per panicle was divided into three parts—apical parts of panicle (APP), median parts (MPP), and basal parts (BPP)—by average distribution (e.g., 11 branches: 4, 3, 4; 13 branches: 4, 5, 4) (**Figure 1C**). Sterile line grain setting was evaluated by categorizing the panicle grains into four distinct types using a light-transmitting plate: filled grains (hard and completely opaque), empty grains (flat and fully translucent, no content inside glumes), blighted grains (flat and partially translucent, residual opaque undeveloped ovaries inside glumes), and infection grains (glumes showing pathological characteristics). Statistical analysis assessed the grain-setting performance.


Grain filling rate (GFR) (%)=Filled grains count/Spikelets

Grain blighted rate (GBR) (%)=Empty grains count/Spikelets

Grain empty rate (GER) (%)=Blighted grains count/Spikelets

Grain infection rate (GIR) (%)=Infection grains count/Spikelets

$$\begin{array}{c} \text{Fertilization success rate(FSP) }\left(\%\right)\\ =\text{(Filled grains count}+\text{empty grains count}\\ +\text{infection filled grains count)}/\text{Spikelets} \end{array}$$

#### 2.3.4 Seed production yield and its components

Sampling followed the average panicle number-based method: three hills were selected from each zone (NR, CR, and FR) per sterile line treatment, with three replicates. Spikelets per panicle and 1,000-grain weight were measured. Sterile lines from each plot were individually harvested, air-dried, and weighed. Yield per unit area (YUA) and equivalent monoculture yield per hectare (EMYH: to account for the land proportion occupied by the restorer and sterile lines, the seed yield of the sterile line plots was converted into equivalent monoculture yield per hectare) were calculated after adjusting the grain moisture content to 13.5%.


$$\begin{array}{c} \text{EMYH }\left({\text{kg ha}}^{-1}\right)=\text{(Sterile linemeasuredyield}/\text{total occupied }\\ \text{area of the field under a single row-ratio treatment)}\times 1000 \end{array}$$

### 2.4 Statistical analysis

Data were analyzed using analysis of variance (ANOVA) after checking assumptions of homogeneity of variances and normality. Means were compared using the least significant difference (LSD) test at the 0.05 probability level with SPSS 23.0 (Statistical Product and Service Solutions Inc., Chicago, IL, USA). RStudio 2023 (Posit PBC, Boston, MA, USA) and Adobe Illustrator 2022 (Adobe Inc., San Jose, CA, USA) were used for figure preparation.

## 3 Results

### 3.1 Effects of row-ratio configuration on the flowering dynamics of parental lines

**Figure 2:**
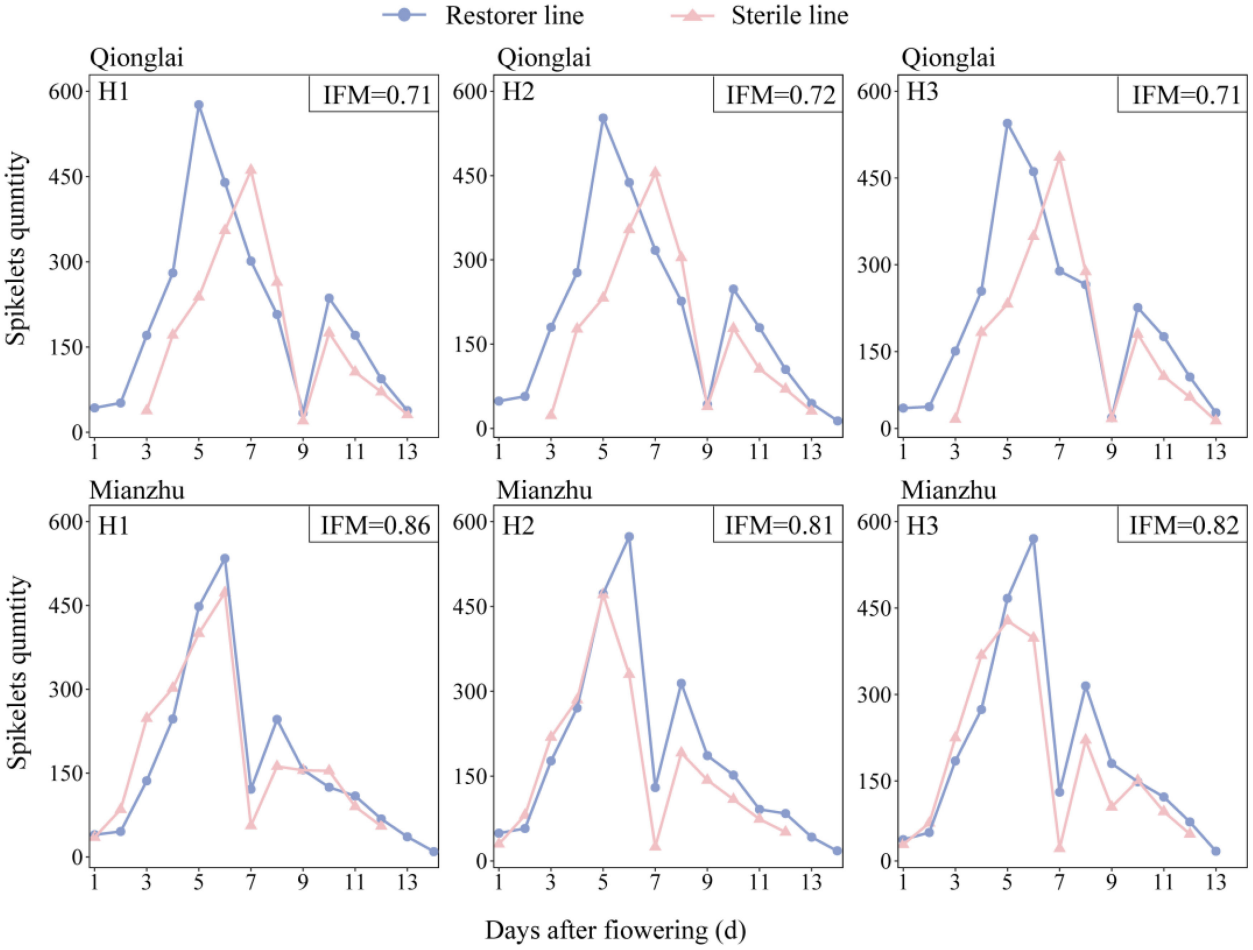
Parent flower encounter status under different row-ratio configuration in Qionglai and Mianzhu. IFM represents index of flowers meeting. H1, H2, and H3 refer to the different row-ratio configuration treatments (18:6, 24:6, and 30:6, respectively).

The effects of different parental row-ratio configurations on the flowering dynamics of the sterile line were not pronounced. With expanding row-ratio, the duration of the sterile line flowering period remained relatively stable, showing a consistent trend across treatments. At two ecological sites (Qionglai and Mianzhu), the flowering duration of sterile lines under all treatments ranged from 11 to 12 days (**Figure 2**). To further assess the synchrony between the parental lines, daily flowering counts of sterile and restorer lines were recorded, and IFM was calculated. A higher IFM value indicates a greater overlap of parental flowering periods, thereby facilitating hybrid rice seed production. The results showed that the IFM ranged from 0.71 to 0.72 at Qionglai and from 0.81 to 0.86 at Mianzhu across treatments, respectively. These findings suggest good flowering synchrony between parents under all row-ratio configurations, with synchrony at Mianzhu being superior to that at Qionglai.

### 3.2 Effects of row-ratio configuration on pollen dispersal across regions of the sterile line

**Figure 3:**
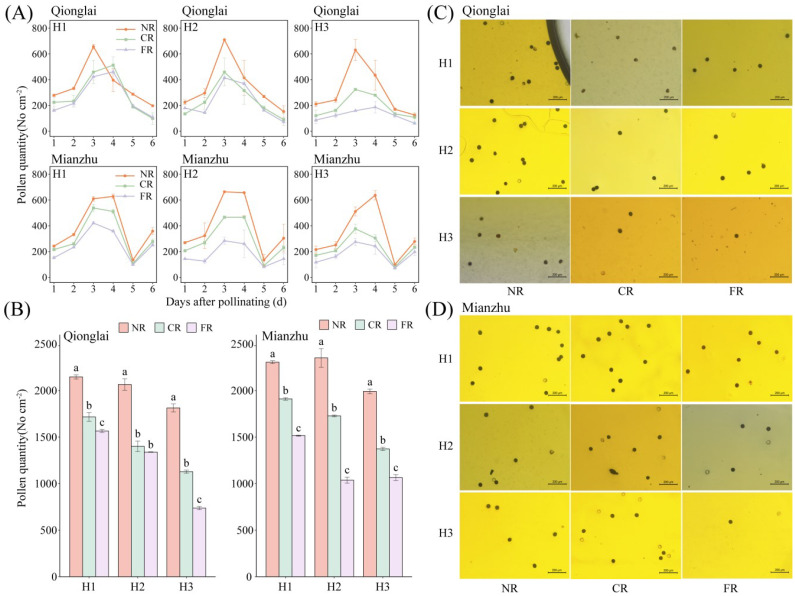
Effects of different row-ratio configurations on pollen quantity distribution between regions of sterile lines. **(A–D)** represent the daily dynamics of pollen quantity between regions of sterile lines. Comparison of pollen total between regions of sterile lines. Spatial distribution of pollen grains per microscopic field across regions in Qionglai and Mianzhu, respectively. H1, H2, and H3 refer to the different row-ratio configuration treatments (18:6, 24:6, and 30:6, respectively). NR, CR, and FR represent near region, central region, and far region, respectively. Different lowercase letters under the same sites mean a significant difference between regions of each treatment at *p <*0.05.

At two ecological sites, the temporal dynamics of pollen quantity across regions followed a similar trend, which was characterized by an initial increase followed by a decline as pollination progressed (with the exception of day 5 at the Mianzhu site, when adverse weather conditions interfered) (**Figure 3A**). Peak pollen abundance occurred on days 3 and 4. With increasing row-ratio, the total pollen quantity consistently exhibited a spatial gradient of NR > CR > FR (**Figure 3B**). Compared with the NR under the H1, H2, and H3 treatments, pollen abundance in the CR and FR at the Qionglai site decreased by 20.06%–27.04% (H1), 32.13%–35.17% (H2), and 37.81%–38.10% (H3), respectively. At the Mianzhu site, the reductions were 17.12%–34.24% (H1), 26.50%–55.87% (H2), and 31.08%–46.54% (H3), respectively. Collectively, these findings indicate that pollen quantity in the sterile line decreases inversely with distance from the restorer line, with the effect being most pronounced under the H3 treatment. This pattern is attributable to the wider block width of the sterile line in H3, which limited pollen deposition on its border rows.

### 3.3 Effects of row-ratio configuration on pollen dispersal across panicle layers of the sterile line

**Figure 4:**
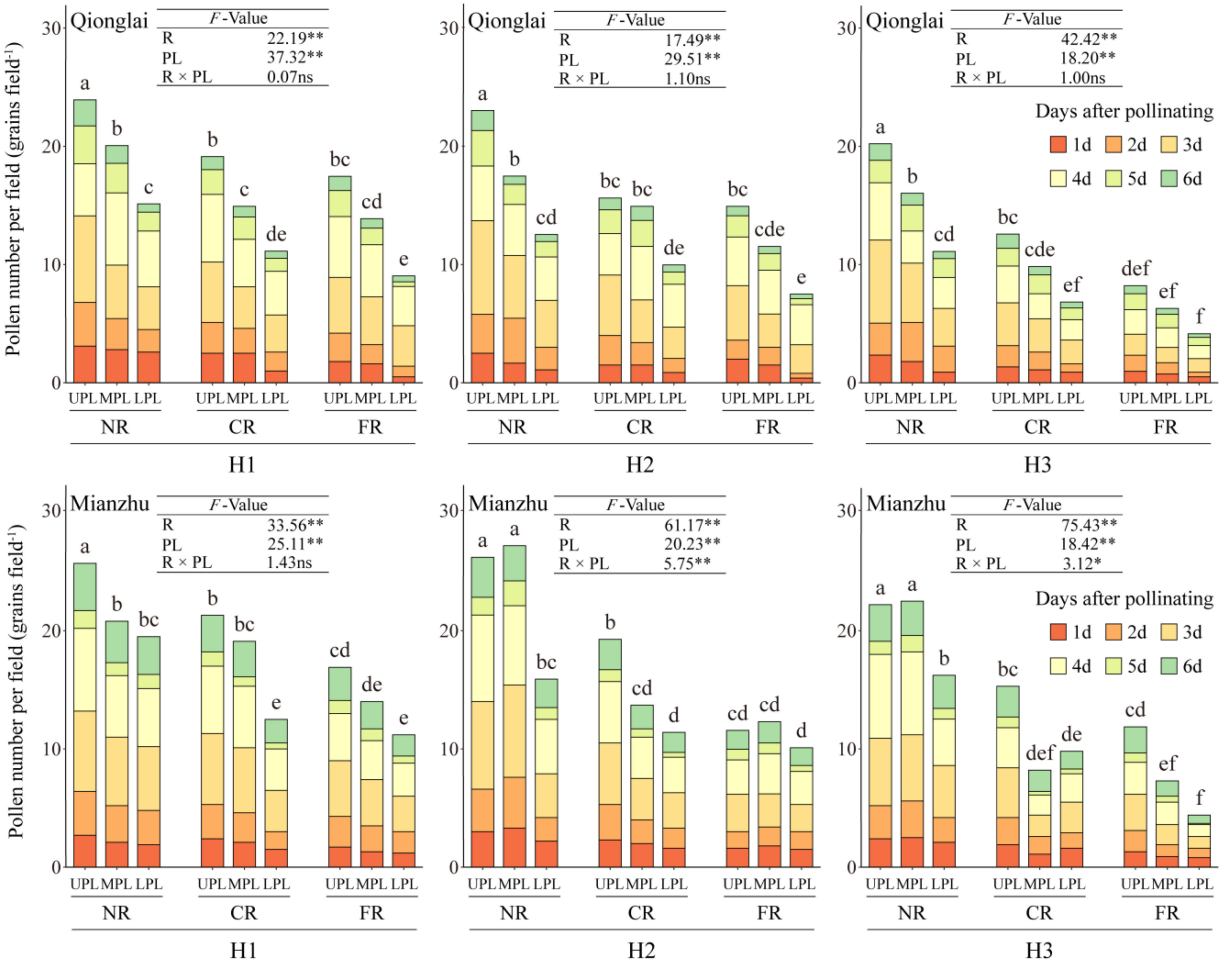
Effects of different row-ratio configurations on panicle layers in pollen quantity of sterile lines in Qionglai and Mianzhu. H1, H2, and H3 refer to the different row-ratio configuration treatments (18:6, 24:6, and 30:6, respectively). R and PL represent the region and panicle layer under the treatment, respectively. NR, CR, FR, ULP, MLP, and LPL represent near region, central region, far region, upper panicle layer, middle panicle layer, and lower panicle layer, respectively. Different lowercase letters under the same site indicate a significant difference in panicle layers among various regions of the same treatment at *p <*0.05. ANOVA *p*-values and symbols are defined as *p < 0.05; **p < 0.01; ns, *p* > 0.05.

Row-ratio configuration significantly influenced the total amount of pollen received by panicles of the sterile line. Across treatments, the total pollen quantity within the NR, CR, and FR generally decreased with panicle position, following the order UPL > MPL > LPL. At the Mianzhu site, however, this vertical gradient was observed only under the H1 treatment (**Figure 4**).

At the Qionglai site, compared with the UPL, pollen quantity in the MPL and LPL of the NR decreased by 16.16%–24.07% and 36.77%–45.33%, respectively; that in the CR by 4.48%–21.96% and 36.21%–41.80%; and that in the FR by 20.51%–23.36% and 48.20%–49.72%, respectively. At the Mianzhu site under H1, pollen deposition in the MPL and LPL of the NR, CR, and FR declined by 19.07% and 24.12%, 10.33% and 41.31%, and 17.16% and 33.73%, respectively.

During peak flowering (day 3 of pollination), the mean pollen number in the NR exceeded three grains per field of view under all treatments. However, at the Qionglai site, pollen deposition did not reach this threshold in the LPL of the CR under H2, the MPL and LPL of the FR under H2, and the MPL and LPL of both the CR and FR under H3. At the Mianzhu site, pollen deposition was also insufficient in the MPL and LPL of the FR under H2 and in the MPL and LPL of the CR and FR under H3.

In summary, pollen quantity in the UPL was sufficient to meet the requirements of hybrid rice seed production under all row-ratio configurations (H1, H2, and H3). However, the LPL under H2 and both the MPL and LPL under H3 failed to meet the minimum daily pollen requirement, potentially constraining fertilization success.

### 3.4 Effects of row-ratio configuration on grain types across regions of the sterile line

**Figure 5:**
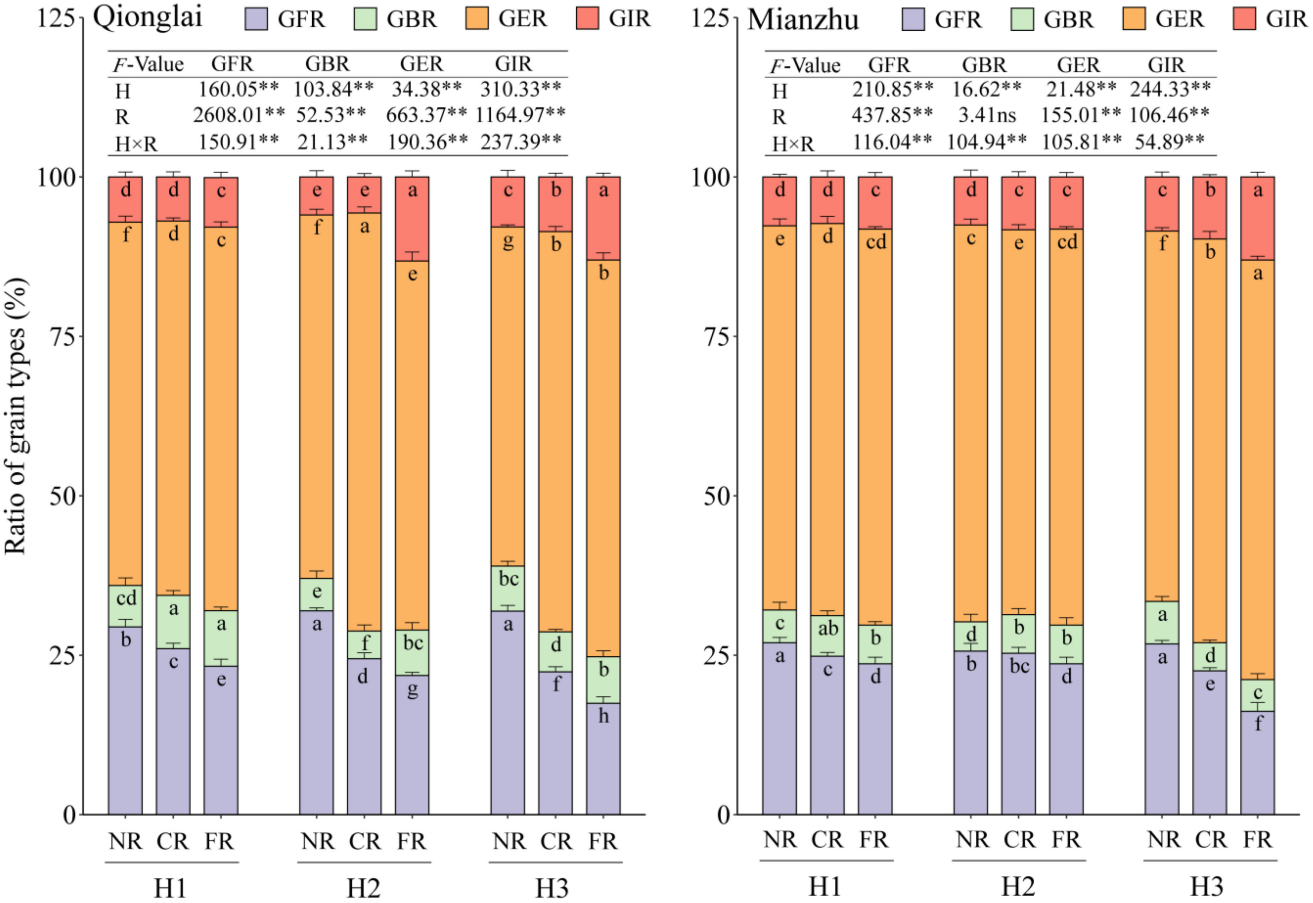
Effects of different row-ratio configurations on grain types of sterile lines between regions. H1, H2, and H3 refer to the different row-ratio configuration treatments (18:6, 24:6, and 30:6, respectively). R represents the region under the treatment. NR, CR, and FR represent near region, central region, and far region, respectively. GFR, GBR, GER, and GIR represent grain filling rate, grain blighted rate, grain empty rate, and grain infection rate, respectively. Different lowercase letters under the same site indicate significant differences between regions of the same treatment (*p* < 0.05). ANOVA *p*-values and symbols are defined as *p < 0.05; **p < 0.01; ns, *p* > 0.05.

As the row-ratio increased, the GFR of the sterile line gradually declined, while the GIR gradually increased; the GBR first decreased and then increased (H1 > H3 > H2). In contrast, the response of GER to row-ratio varied between the two ecological sites. Across treatments, GFR consistently exhibited a spatial gradient of NR > CR > FR, whereas the GER generally followed the opposite trend of FR > CR > NR, with the exception of the H2 treatment at the Qionglai site. By contrast, the GBR displayed only minor fluctuations among regions, with more complex and inconsistent trends (**Figure 5**).

The GFR decline and GER increase from the NR to the distant regions (CR and FR) were amplified under wider row-ratios. This effect was most severe under H3, where GFR in the FR dropped by up to 45.25% at Qionglai and 39.47% at Mianzhu. For GIR, the FR consistently showed higher values than the NR and CR under the H3 treatment at two sites as well as under the H2 treatment at the Qionglai site. In contrast, regional differences were relatively small under H1 and under H2 at the Mianzhu site. Specifically, at Qionglai, GIR in the FR increased by 120.99%–133.07% under H2 and by 51.97%–65.97% under H3 relative to the NR and CR. At Mianzhu, the corresponding increases under H3 were 33.76%–53.29%.

Taken together, these results indicate that altering the row-ratio exerts its influence on the interrelationships among GFR, GIR, and GER in sterile lines primarily by increasing the spatial distance between the sterile and restorer lines.

### 3.5 Effects of row-ratio configuration on grain types across parts of panicle of the sterile line

Under different row-ratio treatments, the spatial pattern of GFR across parts of panicle varied between the two ecological sites. At the Qionglai site, GFR declined progressively from the apical to the basal parts of panicle (APP > MPP > BPP). In contrast, at the Mianzhu site, GFR followed a parabolic trend, peaking in the MPP and declining toward both the APP and BPP (MPP > APP > BPP). In comparison, the patterns of GIR, GBR, and GER were more variable and showed inconsistent trends across sites (**Tables 1**, **2**).

**Table 1:** Effects of row-ratio configuration on seed-setting characteristics of the sterile lines.

Ecological site	Region	Parts of panicle	GFR (%)			FSR (%)		
			H1	H2	H3	H1	H2	H3
Qionglai	Near	Apical	31.37 ± 1.14a	33.88 ± 0.08a	35.27 ± 1.14a	44.15 ± 0.98a	50.38 ± 1.47a	47.48 ± 0.82a
		Median	30.35 ± 1.22b	32.46 ± 0.33b	31.50 ± 0.57b	37.87 ± 1.63b	45.66 ± 0.73b	43.38 ± 0.41b
		Basal	26.66 ± 1.14d	29.61 ± 0.98c	29.02 ± 0.98c	31.91 ± 0.24e	35.50 ± 0.24d	35.09 ± 0.41c
	Central	Apical	27.53 ± 0.82c	26.65 ± 1.06d	24.07 ± 0.57d	36.99 ± 1.39b	36.85 ± 0.73c	34.16 ± 1.14c
		Median	26.30 ± 0.82d	26.00 ± 0.90d	23.22 ± 0.41d	34.13 ± 1.47cd	36.11 ± 0.08cd	29.12 ± 1.47d
		Basal	24.31 ± 0.90f	20.78 ± 0.82e	19.94 ± 1.39f	33.18 ± 1.06d	32.81 ± 1.55e	27.27 ± 0.16e
	Far	Apical	25.82 ± 1.14e	28.86 ± 0.16c	21.08 ± 1.55e	35.16 ± 0.65c	33.67 ± 0.33e	28.69 ± 1.47d
		Median	25.38 ± 1.39e	20.89 ± 0.41e	17.25 ± 0.33g	34.08 ± 0.16cd	30.66 ± 0.98f	25.69 ± 0.82f
		Basal	18.61 ± 0.82g	15.75 ± 0.90f	14.11 ± 1.22h	28.23 ± 0.73f	23.56 ± 1.47g	20.60 ± 0.41g
	Mean		26.26	26.10	23.94	35.08	36.13	32.39
*F*-value	Region		1,290.60**	1,161.06**	1,598.60**	166.97**	1,013.70**	1,935.41**
	Parts of panicle		974.99**	632.26**	247.92**	323.50**	469.11**	533.49**
	R × PP		75.57**	93.38**	8.68**	40.72**	47.74**	28.12**
Mianzhu	Near	Apical	25.29 ± 0.08c	24.56 ± 1.22cd	24.01 ± 0.82b	34.13 ± 1.63cd	34.03 ± 0.08c	34.74 ± 0.49b
		Median	34.42 ± 1.22a	32.18 ± 1.55a	32.61 ± 0.73a	42.44 ± 0.24a	43.78 ± 1.22a	37.52 ± 0.90a
		Basal	21.18 ± 1.14ef	20.19 ± 0.90f	23.72 ± 0.08bc	28.85 ± 0.65g	28.62 ± 1.47e	31.98 ± 0.98c
	Central	Apical	25.14 ± 1.06c	25.17 ± 0.57c	22.7 ± 0.57cd	34.51 ± 0.90c	34.14 ± 0.98c	29.85 ± 1.14d
		Median	27.31 ± 0.41b	26.37 ± 0.98b	22.86 ± 0.16cd	36.21 ± 0.00b	35.32 ± 0.90b	29.76 ± 0.82d
		Basal	22.09 ± 0.33e	24.39 ± 1.22d	22.08 ± 0.73d	32.05 ± 0.98e	32.21 ± 0.65d	27.38 ± 0.49e
	Far	Apical	23.76 ± 1.06d	23.62 ± 0.82e	15.91 ± 1.22f	30.43 ± 0.24f	33.30 ± 0.33c	25.25 ± 1.39f
		Median	26.50 ± 0.73b	24.78 ± 0.90cd	17.91 ± 1.47e	37.26 ± 0.82b	35.98 ± 0.82b	26.57 ± 1.22e
		Basal	20.77 ± 1.31f	19.40 ± 0.90g	14.81 ± 1.47g	33.27 ± 0.08d	27.86 ± 0.33e	20.60 ± 0.00g
	Mean		25.16	24.52	21.85	34.35	33.92	29.29
*F*-value	Region		84.12**	204.47**	659.55**	12.00**	70.15**	894.37**
	Parts of panicle		496.29**	763.88**	122.08**	311.38**	567.28**	180.21**
	R × PP		59.74**	178.99**	48.30**	70.86**	100.42**	12.92**

H1, H2, and H3 refer to the different row-ratio configuration treatments (18:6, 24:6, and 30:6, respectively). R and PP represent the region and parts of panicle under the treatment, respectively. GFR and FSR represent grain filling rate and fertilization success rate, respectively. Different lowercase letters under the same site indicate significant differences among panicles in various regions of the same treatment. ANOVA *p*-values and symbols were defined as follows: *p < 0.05; **p < 0.01; ns, *p* > 0.05.

**Table 2:** Effects of different row-ratio configurations on grain types between parts of panicle of the sterile lines.

Ecological site	Region	Parts of panicle	GIR (%)			GBR (%)			GER (%)		
			H1	H2	H3	H1	H2	H3	H1	H2	H3
Qionglai	Near	Apical	6.83 ± 0.33cd	3.35 ± 0.65h	4.88 ± 1.06g	9.69 ± 1.63ab	5.45 ± 0.82cd	8.01 ± 0.24b	52.12 ± 2.29e	57.32 ± 0.08e	51.84 ± 0.08f
		Median	8.17 ± 0.33b	8.75 ± 1.39d	6.67 ± 0.98f	6.23 ± 0.82c	5.83 ± 1.06c	7.37 ± 0.49bc	55.25 ± 0.16d	52.96 ± 1.39f	54.46 ± 0.73d
		Basal	6.32 ± 1.63de	5.80 ± 0.90f	11.98 ± 1.06c	3.54 ± 1.14d	3.94 ± 1.63e	5.80 ± 1.55de	63.48 ± 0.41a	60.64 ± 1.22d	53.20 ± 0.16e
	Central	Apical	6.88 ± 0.90cd	4.21 ± 0.57g	8.72 ± 1.14d	9.72 ± 0.08a	5.01 ± 1.22d	8.17 ± 0.16b	55.87 ± 1.14d	64.13 ± 1.06c	59.04 ± 1.47c
		Median	6.03 ± 0.16de	5.23 ± 0.41f	7.56 ± 0.16e	6.57 ± 0.49c	3.12 ± 0.57f	4.01 ± 0.41f	61.10 ± 0.24b	65.65 ± 0.57b	65.20 ± 0.73a
		Basal	7.80 ± 1.31bc	7.53 ± 0.65e	9.41 ± 0.49d	8.80 ± 1.63ab	4.86 ± 1.06d	6.57 ± 0.65cd	59.09 ± 0.08c	66.83 ± 1.22a	64.08 ± 0.16a
	Far	Apical	5.29 ± 1.06e	12.96 ± 0.49b	7.06 ± 0.57ef	8.42 ± 0.73b	9.38 ± 1.55a	9.53 ± 1.63a	60.47 ± 0.41bc	56.78 ± 1.47e	62.33 ± 1.47b
		Median	9.75 ± 1.14a	16.42 ± 0.98a	15.24 ± 0.41b	8.62 ± 0.49ab	3.84 ± 1.14ef	7.66 ± 1.06bc	56.24 ± 0.98d	50.88 ± 1.14g	59.85 ± 0.98c
		Basal	8.24 ± 0.24b	10.17 ± 1.39c	16.75 ± 0.82a	9.18 ± 0.41ab	8.12 ± 0.82b	4.68 ± 0.08ef	63.69 ± 1.06a	65.96 ± 1.63ab	64.45 ± 0.90a
	Mean		7.26	8.27	9.81	7.86	5.51	6.87	58.59	60.13	59.38
*F*-value	Region		4.17*	812.014**	390.214**	9.96**	118.04**	40.677**	30.85**	573.34**	621.22**
	Parts of panicle		14.76**	127.71**	422.32**	51.38**	128.04**	38.110**	117.42**	421.93**	46.48**
	R × PP		16.34**	85.41**	134.37**	37.49**	229.04**	53.030**	53.50**	109.98**	40.20**
Mianzhu	Near	Apical	7.11 ± 0.65c	8.09 ± 1.31bc	9.09 ± 1.06cd	4.91 ± 0.08cd	5.11 ± 0.57cd	5.82 ± 1.06cd	62.68 ± 0.82c	62.24 ± 0.82bc	61.08 ± 0.41d
		Median	9.42 ± 0.08b	6.52 ± 1.31d	8.26 ± 1.06cd	4.55 ± 1.31d	4.02 ± 0.57e	5.22 ± 1.63de	51.60 ± 1.47g	57.28 ± 0.98f	53.91 ± 0.65f
		Basal	6.49 ± 0.49cd	7.97 ± 0.65bc	8.13 ± 0.16d	5.96 ± 0.90c	4.69 ± 0.73d	8.97 ± 0.57a	66.38 ± 0.98a	67.14 ± 0.98a	59.18 ± 0.57e
	Central	Apical	6.54 ± 0.82cd	8.67 ± 1.47b	9.22 ± 0.33cd	7.26 ± 0.08b	8.75 ± 1.14b	2.07 ± 0.33g	61.05 ± 1.63d	57.41 ± 1.22f	66.02 ± 1.55b
		Median	5.93 ± 0.73d	8.84 ± 0.33b	10.68 ± 0.16b	3.00 ± 1.47e	4.18 ± 0.82e	3.63 ± 1.06f	63.77 ± 0.65b	60.61 ± 0.82d	62.82 ± 0.82c
		Basal	9.42 ± 1.31b	7.37 ± 0.65cd	9.31 ± 0.57c	8.90 ± 1.14a	5.32 ± 0.65c	7.54 ± 1.31b	59.59 ± 1.06e	62.92 ± 0.41b	61.07 ± 1.22d
	Far	Apical	6.60 ± 1.22cd	10.27 ± 0.82a	10.61 ± 0.41b	3.23 ± 0.73e	4.16 ± 1.06e	6.70 ± 0.98bc	66.40 ± 0.33a	61.95 ± 1.47c	66.78 ± 0.33ab
		Median	10.50 ± 0.08a	10.95 ± 1.39a	13.88 ± 0.24a	7.14 ± 0.82b	5.55 ± 0.65c	4.78 ± 0.90e	55.86 ± 0.73f	58.72 ± 0.82e	63.43 ± 0.08c
		Basal	7.46 ± 0.82c	11.09 ± 0.73a	14.58 ± 1.55a	7.8 ± 0.41b	9.48 ± 0.57a	3.45 ± 0.24f	63.98 ± 0.16b	60.03 ± 0.49d	67.15 ± 1.39a
	Mean		7.72	8.86	10.42	5.86	5.70	5.35	61.26	60.92	62.38
*F*-value	Region		6.49**	102.56**	134.82**	30.85**	573.34**	621.22**	23.27**	69.59**	363.98**
	Parts of panicle		28.25**	0.57ns	11.66**	117.42**	421.93**	46.48**	339.88**	283.66**	122.21**
	R × PP		40.20**	9.28**	16.54**	53.50**	109.97**	40.20**	236.60**	175.20**	33.42**

H1, H2, and H3 refer to the different row-ratio configuration treatments (18:6, 24:6, and 30:6, respectively). R and PP represent the region and parts of panicle under the treatment, respectively. GIR, GBR, and GER represent grain empty rate, grain blighted rate, and grain empty rate, respectively. Different lowercase letters under the same site indicate significant differences among panicles in various regions of the same treatment. ANOVA *p*-values and symbols are defined as *p <no><</no> 0.05; **p <no><</no> 0.01; ns, *p* > 0.05.

At the Qionglai site, relative to the APP, GFR under different row-ratio treatments decreased by 3.24%–10.69% and 12.59%–17.72% in the MPP and BPP of the NR, by 2.46%–4.49% and 11.70%–22.04% in the MPP and BPP of the CR, and by 1.70%–27.62% and 27.93%–45.42% in the MPP and BPP of the FR. At the Mianzhu site, compared with the MPP, GFR decreased by 23.68%–26.53% (APP) and 27.25%–38.48% (BPP) in the NR, by 0.73%–7.94% (APP) and 3.40%–19.11% (BPP) in the CR, and by 4.70%–11.16% (APP) and 17.29%–21.72% (BPP) in the FR. For GIR, fluctuations across parts of panicle were relatively minor and inconsistent in both the NR and CR, but the FR consistently exhibited higher diseased grain rates than the other two region.

Taken together, these findings suggest that row-ratio configuration significantly influences the spatial distribution of pollen supply, thereby affecting grain type composition in the sterile line. Consequently, optimizing row-ratio requires not only improving pollen utilization efficiency but also ensuring the coordinated development of spikelet fertilization and grain filling across panicle positions.

### 3.6 Effects of row-ratio configuration on seed-setting characteristics of the sterile line

Row-ratio configuration exerted a significant influence on the seed-setting characteristics of the sterile line across regions and parts of panicle. GFR declined progressively with the expansion of row-ratios (H1 > H2 > H3). FSR, however, showed divergent trends across ecological sites: at the Qionglai site, it first increased and then decreased (H2 > H1 > H3), whereas at the Mianzhu site it declined steadily (H1 > H2 > H3). Notably, the performance of FSR across regions and parts of the panicle was largely consistent with that of GFR (**Table 1**).

Across different row-ratio treatments, the distribution patterns of FSR in different parts of the panicle varied by site. At the Qionglai site, FSR decreased progressively with descending parts of the panicle (APP > MPP > BPP). In contrast, at the Mianzhu site, it followed a parabolic pattern (MPP > APP > BPP), except for CR under the H3 treatment. Specifically, at Qionglai, FSR in the NR were 8.65%–14.23% and 26.11%–29.54% lower in the MPP and BPP compared with the APP; in the CR, the corresponding reductions were 2.02%–14.75% and 10.30%–20.17% and in the FR were 3.08%–10.44% and 19.72%–30.02%, respectively. At the Mianzhu site, relative to the MPP, FSR in the NR decreased by 7.43%–22.27% (APP) and 14.79%–34.62% (BPP); in the CR by -0.31%–4.70% (APP) and 7.98%–11.49% (BPP); and in the FR by 4.99%–18.33% (APP) and 10.72%–22.58% (BPP).

The highest mean FSR values were observed in the APP of the NR under H2 at the Qionglai site (50.38%) and in the MPP of the NR under H2 at the Mianzhu site (43.78%). However, GFR at these corresponding positions were reduced by 32.75% and 26.49%, respectively. Across all treatments, the GFR values were consistently and substantially lower than FSR, indicating that successful pollination of the sterile line does not necessarily guarantee effective grain development.

### 3.7 Effects of row-ratio on seed production yield and its components

Row-ratio configuration significantly influenced hybrid seed production and its yield components (**Table 3**). With the expansion of row-ratios, both GFR and YUA declined (H1 > H2 > H3). In contrast, 1,000-grain weight either increased progressively (Qionglai: H3 > H1 > H2) or followed a U-shaped trend (Mianzhu: H3 > H1 > H2). The panicle number and spikelets per panicle showed no significant differences among treatments. Under the H1 treatment, YUA at the Qionglai and Mianzhu sites were 2,107.50 and 2,201.62 kg ha^−1^, respectively, representing increases of 4.18% and 3.18% compared with H2 and 6.74% and 5.77% compared with H3. Similarly, GFR under H1 was 0.62% and 9.69% higher than under H2 and 3.24% and 15.87% higher than under H3 at Qionglai and Mianzhu, respectively. Interestingly, the EMYH value increased with wider row-ratios (H3 > H2 > H1). Under the H3 treatment, EMYH at Qionglai and Mianzhu reached 1,645.43 and 1,734.59 kg ha^−1^, which were 4.10% and 1.68% higher than H1 and 5.05% and 1.62% higher than H2, respectively.

**Table 3:** Effects of different row-ratio configurations on yield and its component of seed production.

Ecological site	Treatment	Panicle number (×10^4^ ha^-1^)	Spikelets per panicle	1,000-grain weight (g)	GFR (%)	YUA (kg ha^-1^)	EMYH (kg ha^-1^)
Qionglai	H1	300.46 ± 6.16a	133.41 ± 2.93a	27.24 ± 0.06b	26.26 ± 0.35a	2,107.50 ± 20.83a	1,580.62 ± 15.62b
	H2	306.93 ± 10.80a	134.07 ± 1.67a	26.87 ± 0.14c	26.10 ± 0.79a	2,022.85 ± 9.66b	1,618.28 ± 7.73a
	H3	289.43 ± 12.59a	135.02 ± 1.86a	27.49 ± 0.06a	23.94 ± 0.20b	1,974.51 ± 18.26c	1,645.43 ± 15.22a
*F*-value	H	4.70ns	0.28ns	37.96**	12.23*	32.32**	12.86*
Mianzhu	H1	287.59 ± 2.35a	136.96 ± 3.71a	27.28 ± 0.06b	25.31 ± 0.28a	2,201.62 ± 23.81a	1,651.22 ± 17.86b
	H2	288.31 ± 6.54a	135.94 ± 2.48a	27.34 ± 0.15b	24.52 ± 0.48b	2,133.69 ± 7.56b	1,706.95 ± 6.05a
	H3	296.09 ± 3.09a	132.07 ± 2.55a	28.01 ± 0.09a	21.85 ± 0.33c	2,081.51 ± 5.07c	1,734.59 ± 4.23a
*F*-value	H	0.18ns	1.43ns	21.05**	160.34**	16.52*	27.66**

H1, H2, and H3 refer to the different row-ratio configuration treatments (18:6, 24:6, and 30:6, respectively). GFR, YUA, and EMYH represent grain filling rate, yield per unit area, and equivalent monoculture yield per hectare, respectively. Different lowercase letters under the same site indicate significant differences between treatments (*p* < 0.05). ANOVA *p*-values and symbols are defined as *p < 0.05; **p < 0.01; ns, *p* > 0.05.

### 3.8 Linear regression analysis of pollen diffusion effects on outcross setting rate

**Figure 6:**
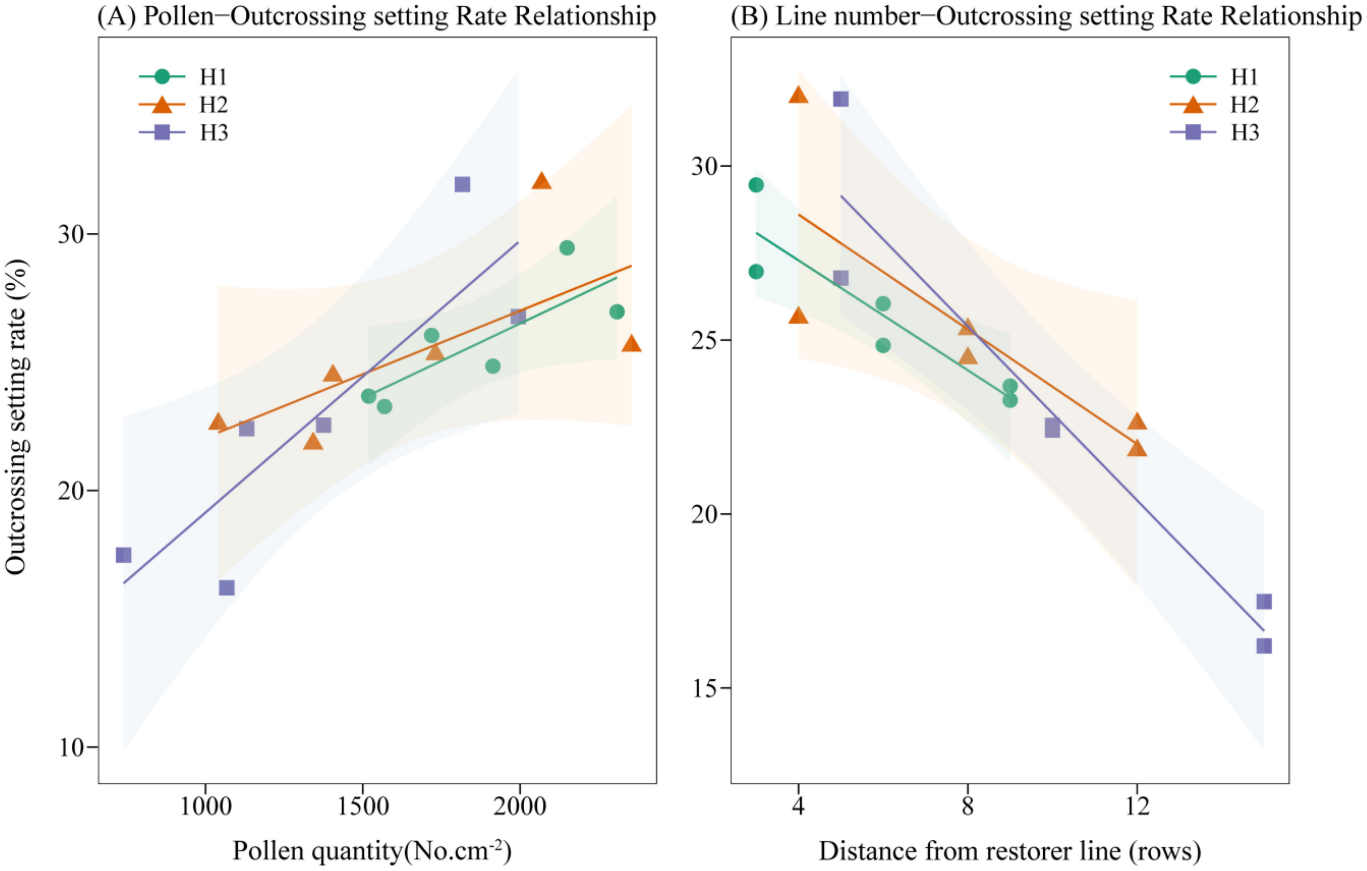
Regression model of pollen deposition and distance decay on outcross setting rate. H1, H2, and H3 refer to the different row-ratio configuration treatments (18:6, 24:6, and 30:6, respectively).


As shown in **Table 4**, the *t*-test results indicated that both the number of sterile rows and pollen quantity had highly significant effects on the dependent variable (outcrossing seed-setting rate), with *P*-values less than 0.01. The standardized regression coefficients were -0.83 and 0.0047, respectively. A linear regression analysis was conducted using the number of sterile rows and pollen quantity as independent variables and the outcrossing seed-setting rate as the dependent variable (**Figure 6**). The resulting model was as follows:

**Table 4:** Results of outcross setting rate linear regression analysis.

Item	Coefficient	SE	*t*	*P*
Constant	41.27	3.45	11.95	0.000
Line number	-0.83	0.13	-6.38	0.000
Pollen quantity	0.0047	0.0012	3.92	0.001
*R* ^2^	0.82			
Adjusted *R*^2^	0.78			
*F*-value	17.23***			

ANOVA *p*-values and symbols were defined as follows: *p < 0.05; **p < 0.01; ***p < 0.001; ns, *p* > 0.05.

Outcrossing seed-settingrate=41.27-0.83×Row number+0.0047×Pollen quantity

The results demonstrated that pollen quantity exerted a significant positive effect on seed-setting, with every additional 100 pollen grains increasing the outcrossing seed-setting rate by 0.47%. In contrast, row distance exhibited a significant negative effect: for every additional row, the outcrossing seed-setting rate decreased by 0.83%. Based on the model’s explanatory power, the two independent variables together accounted for 82.40% of the variation in outcrossing seed-setting rate.

## 4 Discussion

### 4.1 Effects of row-ratio configuration on pollen dispersal and pollination efficacy

Hybrid rice seed production relies on cross-pollination between parental lines, where flowering synchrony and pollination effectiveness directly determine GFR and thus constitute key factors influencing seed yield (Li et al., 2025). Adequate pollen dispersal that fully covers the sterile line area can significantly enhance GFR and increase seed production. The spatial distribution of pollen within the field is jointly regulated by sterile block width, pollination method, and the cross-pollination dynamics between parental lines and represents a crucial mechanism shaping the coexistence of parental lines. Previous studies have shown that AUAV-assisted pollination enhances pollen dispersal distance through airflow, achieving more uniform pollen distribution while minimizing mechanical damage to plants, thereby providing an effective method for large row-ratio configurations (Li et al., 2021; Wang et al., 2022; Xiao et al., 2021). According to Namai Hyoji et al (Namai and Kato, 1987; Yi et al., 2009), ensuring that each stigma of the sterile line receives at least three pollen grains on average is essential to maintain a satisfactory GFR.

**Figure 7:**
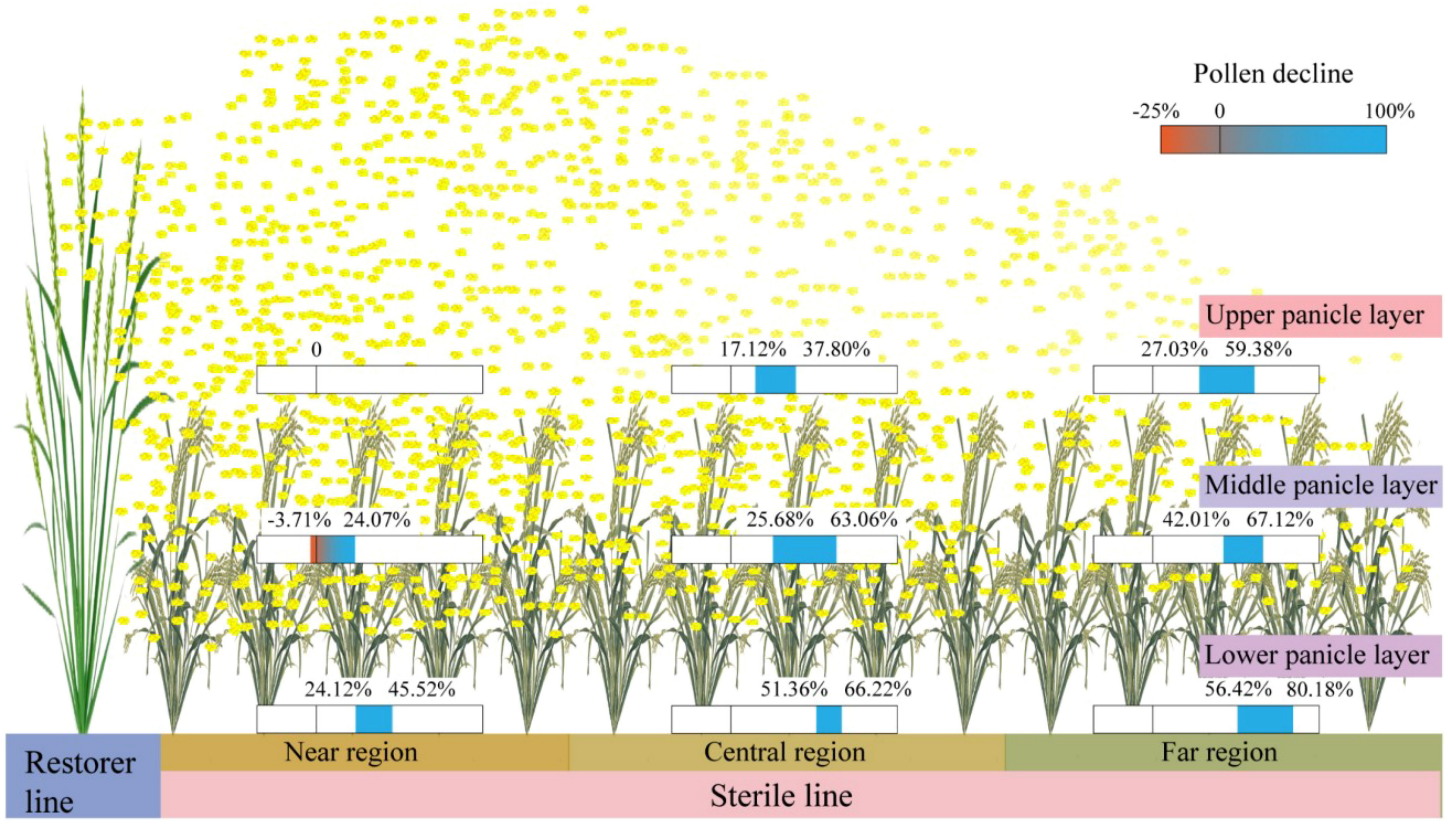
Spatial distribution pattern map of restorer pollen in sterile line regions and panicle layers. Data showing pollen decline across different parts of the sterile line are calculated with reference to the UPL in the NR under the H1 treatment.

In this study, the maximum pollen counts per microscopic field of view in H1, H2, and H3 treatments were observed in the UPL of the NR, with values of 7.13, 7.91, and 7.03 grains at Qionglai and 7.00, 9.00, and 7.10 grains at Mianzhu, respectively. The corresponding values for the LPL were 3.61, 3.41, and 3.19 grains at Qionglai and 4.90, 4.60, and 3.93 grains at Mianzhu. These results indicate that in H1, the narrower sterile block width shortened pollen dispersal distance, enabling effective coverage within sterile rows and resulting in a relatively high GFR in border rows. By contrast, in H3, the wider sterile block width weakened pollen coverage in border rows, with pollen quantities in the UPL reaching the minimum threshold only during peak flowering (**Figure 7**). Given the stochastic nature of cross-pollination in the field, merely meeting the minimum pollen requirement per stigma does not necessarily translate into higher GFR across additional florets, thereby constraining overall seed yield.

Previous studies have demonstrated the distribution of airflow generated by AUAV rotors within crop canopies, revealing that pollen dispersal is positively correlated with the wind field in the x-direction. However, the rotors also generate airflow in the y- and z-directions, with the y-direction prevailing, thereby creating asymmetry and heterogeneity in field pollen distribution (Li et al., 2018; Weng et al., 2023). In this context, optimizing the row-ratio configuration represents a critical agronomic strategy to address the challenges associated with this inherent airflow heterogeneity. An appropriately designed row-ratio, particularly a wider sterile block, can be spatially arranged to leverage the dominant y-axis airflow for enhanced pollen penetration across a greater number of sterile rows, while simultaneously mitigating the negative effects of pollen attenuation along the x-axis (i.e., with increasing distance from the restorer line). Furthermore, the rotor-induced wind field agitates the panicles layer of the sterile line, which reducing the occurrence of “hidden panicles beneath leaves,” thereby improving pollen deposition and utilization in the MPL and LPL.

In the present study, AUAV pollination was conducted by flying parallel to the restorer rows. Under the influence of rotor airflow, pollen from restorer panicles dispersed into the near and central sterile line regions, where the reduction in GFR from the UPL to MPL and LPL of the sterile line was relatively small. By contrast, in the FR, inadequate airflow coverage resulted in a pronounced decline in GFR in the MPL and LPL. This pattern demonstrates that optimal row-ratio configuration transcends merely increasing row numbers; it involves designing a spatial layout that actively leverages the AUAVs’ wind field to maximize effective coverage. Furthermore, the resulting pollen distribution interacts with cross-pollination dynamics—such as leaf architecture, floret opening angle, and stigma exsertion—which can further modulate dispersal and ultimately favor improved GFR (Elshamey et al., 2022).

Overall, an increased pollen supply enhances the probability of pollen–stigma contact, thereby promoting fertilization and elevating GFR. This observation is consistent with previous studies emphasizing the importance of ensuring sufficient pollen availability in seed production (Xu et al., 2015). Nevertheless, increasing distance significantly reduced pollen dispersal efficiency, likely due to airflow dissipation, pollen viability loss, and other constraints during transit, which collectively lowered the number of viable pollen grains reaching stigmas, and ultimately suppressed GFR. These results underscore the need for the rational planting layouts to mitigate the negative effects of distance on pollination success.

### 4.2 Effects of row-ratio configuration on grain-setting characteristics and grain types of sterile lines

A comparison of pollen-shedding dynamics with parental flowering times revealed that the peak pollen availability in the sterile lines coincided precisely with the peak flowering period of the restorer line, indicating that field pollen abundance is closely linked to flowering synchrony and thereby shapes the spatial pattern of seed-setting along the panicle. Previous studies have shown that in hybrid rice, GFR typically follows the order APP > MPP > BPP. This gradient is largely attributable to differences in floret anthesis timing, with spikelets in the upper portion flowering earlier and classified as “dominant florets,” while those in the basal portion flower later and are considered “inferior florets” (Dong et al., 2011; Li, 2021; Wei et al., 2024). The seed-setting distribution of sterile-line parts of panicle in this study exhibited a similar trend, reflecting the influence of dominant versus inferior floret characteristics (Huang et al., 2023). Moreover, positional differences in spikelet receptivity, coupled with the inhibitory effects of dominant grains on the grain filling of inferior ones, further contributed to the spatial variation in seed-setting (Wang et al., 2020; Li, 2000; Yang, 2010).

**Figure 8:**
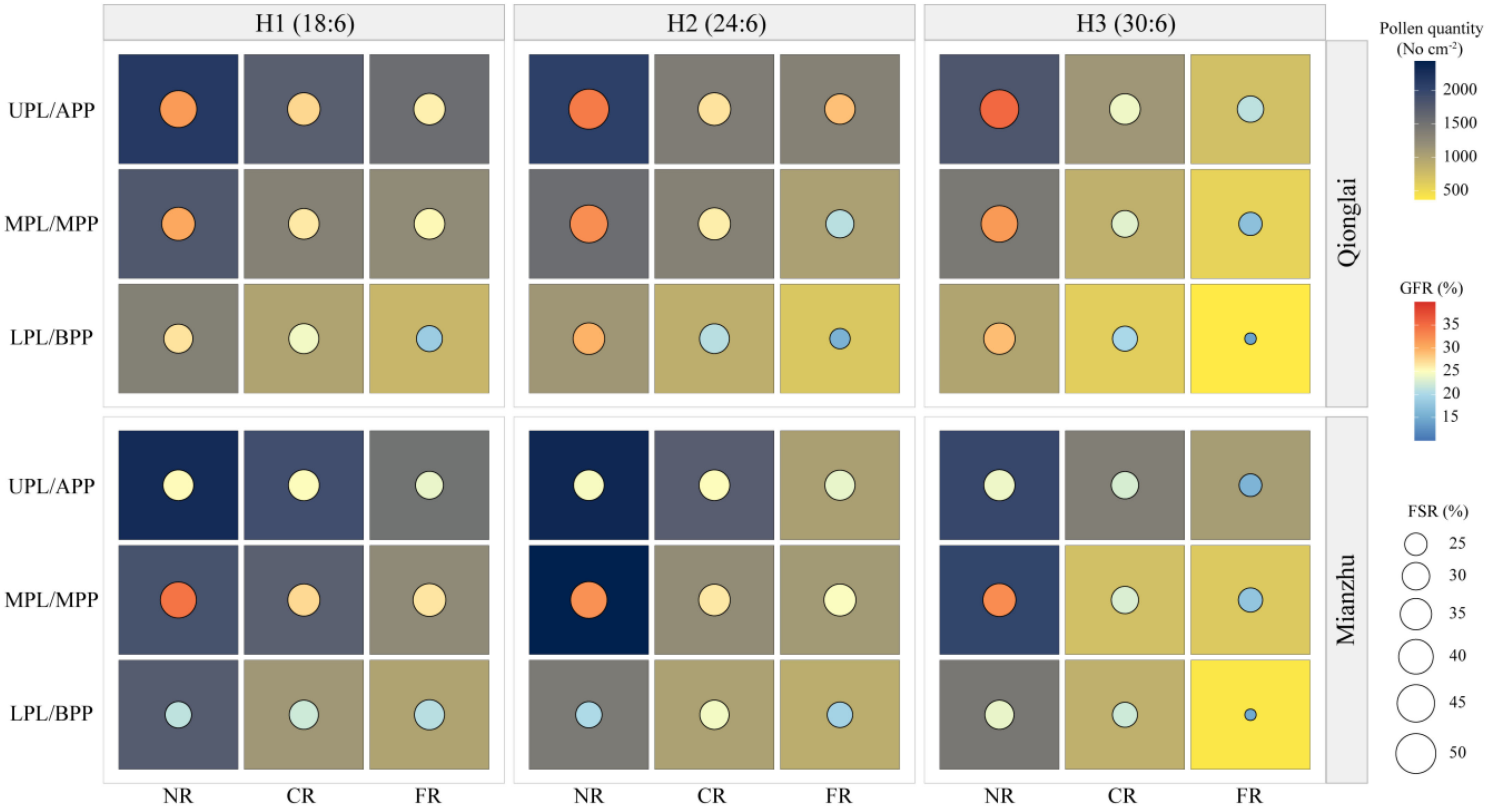
Spatial coupling relationship among pollen quantity, FSR, and GFR.

Our experimental results demonstrated that at the Qionglai site, asynchronous flowering between parental lines ensured sufficient pollen availability during sterile-line anthesis, leading to a panicle GFR pattern of APP > MPP > BPP. In contrast, at the Mianzhu site, flowering synchronization between parental lines resulted in sequential anthesis from the top to the bottom of the panicle, with peak anthesis concentrated in the MPP. Consequently, GFR followed the order MPP > APP > BPP, with only minor differences between the APP and MPP. This may be attributed to the prolonged receptivity of exserted stigmas (approximately 3 days) under conditions of abundant pollen during the peak flowering stage (Gupta et al., 2015), which enhanced pollen utilization efficiency (**Figure 8**). Notably, GFR in the MPP of sterile-line parts of panicles was relatively stable across conditions, whereas the APP and BPP were more strongly influenced by flowering synchrony. Nevertheless, in panicles with overall high GFR, the differences among parts of panicle sections tended to diminish, suggesting that high levels of pollination can buffer positional disparities in GFR.

The configuration of parental row-ratios is not only a critical determinant of GFR in hybrid rice seed production but also shapes the spatiotemporal coordination between pollen dispersal per unit area and stigma availability. In regions or parts of panicles with sufficient pollen, spikelets exhibited higher pollination success and significantly enhanced GFR. Conversely, in FR and BPP where pollen was scarce, GFR declined, the proportion of unpollinated spikelets increased, and consequently, the proportion of GER rose sharply. This distribution pattern closely followed the gradient of pollen availability. The GIR was indirectly related to pollen quantity and closely linked to the duration of spikelet opening in sterile lines, which determines the window for receiving restorer-line pollen (Huang and Zeng, 2025). Sterile lines that received pollen early in anthesis tended to close their spikelets sooner, thereby reducing the risk of pathogen infection. By contrast, sterile lines located farther from the pollen source experienced prolonged stigma exposure due to limited pollen supply, increasing their susceptibility to infection. Previous studies have demonstrated that during rice anthesis, the expression of flowering-related genes is markedly upregulated, whereas the expression of disease-resistance genes is downregulated, making the flowering stage particularly vulnerable to pathogen invasion (Xu et al., 2024; Yi et al., 2024).

Our results showed that GFR in sterile lines decreased progressively from NR to CR to FR, whereas GIR increased, consistent with earlier reports. The formation of unfilled grains (shriveled kernels) exhibited a dual response to pollen quantity. In NR, where pollen quantity was high, the large number of pollinated spikelets combined with stochastic pollination effects and intra-panicle resource competition contributed to elevated sterility. In FR, insufficient pollen quantity limited fertilization success, similarly increasing the GBR. These findings highlight the combined regulatory roles of “pollination base size” and “resource availability for grain filling” in determining spikelet fertility outcomes.

### 4.3 Effects of row-ratio configuration on seed production yield and production efficiency

Variations in parental row-ratios primarily affect outcrossing performance by altering the spatial distance between sterile and restorer lines (Kang et al., 2010) rather than modifying the intrinsic relationships among GFR, GIR, and GER. When the row-ratio was adjusted from 18:6 to 30:6, the increase in sterile-line rows reduced the pollen quantity per unit area, leading to lower GFR per row, accompanied by elevated GIR and GER, thereby decreasing YUA. However, the overall increase in sterile-line rows substantially raised the total number of spikelets and enhanced the aggregate pollen-receiving capacity, which contributed positively to EMYH. This indicates that the expansion of sterile-line rows compensates for the YUA reduction and plays a dominant role in improving total production. In other words, within the effective range of pollen dispersal, expanding the sterile-line planting area is a core strategy to boost overall yield.

Pollen data further demonstrated that, compared with 18:6 and 24:6 configurations, the 30:6 row-ratio was still able to maintain effective pollination. Beyond stabilizing land-use efficiency and simplifying field layout, a wider row-ratio also facilitated mechanized transplanting and harvesting, thereby reducing production costs. Nevertheless, the proportion of unfilled grains in sterile lines remained consistently high across two ecological sites (59.37%–61.52%), in line with previous studies, which was largely attributable to unfertilized spikelets (Long and Shu, 2000).

It is noteworthy that the observed GFR across treatments was consistently lower than the theoretical FSR, primarily due to constraints during grain filling (blighted grains) and disease infection (infection grains). The underlying causes include (1) fertilization barriers, where insufficient pollen supply or environmentally induced declines in pollen or stigma viability result in incomplete fertilization and (2) grain development suppression, where competition for assimilates, hormonal imbalances, and abiotic stresses (e.g., endosperm starch gelatinization caused by high temperatures during the grain filling stage or mold development induced by excessive humidity) lead to endosperm abnormalities and, ultimately, to blighted grains or infection grains (Deng et al., 2021; Wu et al., 2019; Shi et al., 2020). The GIR, averaging around 8% across treatments, not only caused substantial yield losses but also impaired seed quality. In hybrid rice seed production, optimizing the parental row-ratio requires balancing seed-setting efficiency with the reduction of diseased grains. It is also essential to integrate field microclimate, disease resistance, and agronomic practices to minimize panicle diseases and ensure simultaneous improvement in both yield and seed quality.

In conclusion, optimizing the parental row-ratio is fundamentally a process of balancing the sterile line population size against pollen dispersal efficiency. Our findings directly address the core challenge outlined in the introduction: how to design a field configuration compatible with full mechanization while ensuring high seed yield. This study demonstrates that expanding the row-ratio, as exemplified by the H3 (30:6) treatment, effectively increases the number of sterile rows—and thus panicles—per unit area, thereby improving land-use efficiency and aligning sterile block width with standard harvester cutting widths. However, this expansion incurs a trade-off: pollen density diminishes in border rows due to dispersal attenuation, resulting in a lower average seed-setting rate. The higher EMYH achieved under the widest row-ratio indicates that the yield gain from the substantially enlarged sterile line population can, under AUAV-assisted pollination, compensate for and even exceed the losses attributable to reduced seed-setting per row. Therefore, the optimal row-ratio identified in this study represents an equilibrium point at which the benefit of increased panicle number is maximized, while the negative impact of pollen limitation is controlled to an acceptable level. This balance provides a practical and effective field configuration strategy for fully mechanized hybrid rice seed production without compromising yield.

## 5 Conclusion

Different parental row-ratio configurations exerted significant effects on pollen distribution, seed-setting characteristics, and yield performance. Expansion of the row-ratio intensified the decline in pollen quantity both across sterile-line regions and within parts of panicles, which consequently increased the GER and GIR while reducing GFR and YUA. However, the increase in the number of sterile-line rows partially offset the reduction in YUA, thereby enhancing EMYH. Overall, after optimizing pollen utilization efficiency and mitigating the negative effects of distance-dependent pollen attenuation, the 30:6 row-ratio (H3) not only demonstrated higher yield potential but also a simplified field layout, making it more compatible with mechanized operations. For hybrid rice seed production using mechanized transplanting, harvesting, and AUAV-assisted pollination, large parental row-ratios should be employed.

## Data Availability

The original contributions presented in the study are included in the article/**Supplementary Material**. Further inquiries can be directed to the corresponding authors.

## References

[B1] Chen L. Y. Lei D. Y. Tang W. B. Deng H. B. Xiao Y. H. Zhang G. L. (2015). Challenges and strategies of hybrid rice development in China. Hybrid Rice 30, 1–4. doi: 10.16267/j.cnki.1005-3956.201505001

[B2] Deng F. Zeng Y. Li Q. He C. Li B. Zhu Y. . (2021). Decreased anther dehiscence contributes to a lower fertilization rate of rice subjected to shading stress. Field Crops Res. 273, 108291. doi: 10.1016/j.fcr.2021.108291

[B3] Ding C. Luo X. K. Wu Q. Lu B. Ding Y. F. Wang S. H. . (2021). Compact plant type rice has higher lodging and N resistance under machine transplanting. J. Integr. Agric. 20, 65–77. doi: 10.1016/S2095-3119(20)63229-4

[B4] Dong M. H. Xie Y. L. Qiao Z. Y. Liu X. B. Wu X. Z. Zhao B. H. . (2011). Variation in carbohydrate and protein accumulation between spikelets at different positions within a rice panicle during grain filling. Chin. J. Rice Sci. 25, 297–306. doi: 10.3969/j.issn.1001-7216.2011.03.011

[B5] Elshamey E. A. Z. Hamad H. S. Alshallash K. S. Alghuthaymi M. A. Ghazy M. I. Sakran R. M. . (2022). Growth regulators improve outcrossing rate of diverse rice cytoplasmic male sterile lines through affecting floral traits. Plants 11, 1291. doi: 10.3390/plants11101291, PMID: 35631716 PMC9148114

[B6] Gupta R. Sutradhar H. Chakrabarty S. K. Ansari M. W. Singh Y. (2015). Stigmatic receptivity determines the seed set in Indian mustard, rice and wheat crops. Communicative Integr. Biol. 8, e1042630. doi: 10.1080/19420889.2015.1042630, PMID: 27066163 PMC4802787

[B7] He L. X. Luo H. W. Duan M. Y. Kong L. L. Tang X. R. (2022). Mechanized hybrid rice seed production: planting density, the flight height of an unmanned aerial vehicle, fertilizer application, and the row-ratio of parents. Agronomy 12, 1572. doi: 10.3390/agronomy12071572

[B8] Huang G. F. (2015). Effects of different row ratio and density on hybrid rice seed production yield formation (Changsha, Hunan: Master Dissertation of Hunan Agricultural University).

[B9] Huang Y. W. Cheng C. Niu F. Zhou J. H. Chu H. W. Zhang A. P. . (2022). Research status and practice of full-process mechanized seed production in japonica hybrid rice. Hybrid Rice 37, 1–7. doi: 10.16267/j.cnki.1005-3956.20210805.275

[B10] Huang B. C. Tao Y. F. Qin Q. Li H. Zhou Z. L. Guo J. Y. . (2023). Investigation on outcrossing seed-setting characteristics of machine-transplanted hybrid rice seed production. Scientia Agricultura Sin. 56, 3960–3974. doi: 10.3864/j.issn.0578-1752.2023.20.004

[B11] Huang Y. M. Zeng X. C. (2025). Effects of pollination on spikelet closure in rice (*Oryza sativa* L.). J. Anhui Agric. Univ. 52, 212–219. doi: 10.13610/j.cnki.1672-352x.20250519.019

[B12] Jiang R. Lin J. Q. Lin Z. H. Liu A. M. Deng K. H. (2025). Design and operational parameter optimization of endurance-extended drone for supplementary pollination in hybrid rice breeding. Trans. Chin. Soc. Agric. Machinery 56, 229–239. doi: 10.6041/j.issn.1000-1298.2025.02.022

[B13] Kang Y. Q. Li Y. Li K. P. (2010). Occurrence and control of rice kernel smut in seed production of hybrid rice. Yunnan Agric. Sci. Technol. 39, 42–44. doi: 10.3969/j.issn.1000-0488.2010.02.021

[B14] Kong L. B. Lin Q. Nie Y. L. Wang J. J. Wei H. (2023). Current situation and countermeasures analysis of China’s crop seed industry. J. Agric. Sci. Technol. 25, 1–13. doi: 10.13304/j.nykjdb.2022.0507

[B15] Li T. (2000). Research stage and strategy of rice bearing obstacle. J. Sichuan Agric. Univ. 18, 370–373. doi: 10.3969/j.issn.1000-2650.2000.04.022

[B16] Li X. F. (2021). Study on the relationship between material ttransport and grain filling characteristics in large-panicle rice (Hefei, Anhui: Master Dissertation of Anhui Agricultural University).

[B17] Li H. J. He X. K. Song J. L. Yang Y. (2021). Comparison of global R&D of agricultural unmanned aerial vehicle, based on bibliometrics. J. China Agric. Univ. 26, 154–167. doi: 10.11841/j.issn.1007-4333.2021.09.17

[B18] Li J. Y. Lan Y. B. Wang J. W. Chen S. D. Yao W. X. Huang C. . (2018). Distribution law of rice pollen in the wind field of small UAV. Agric. Sci. Eng. China 30 (2) 13-19, 36. doi: 10.3969/j.issn.1002-5103.2018.02.003

[B19] Li Z. Y. Li W. Chen G. Y. Li T. Zhu C. H. Deng S. J. . (2025). Effects of different gibberellin treatments on flowering dynamics and outcrossing characteristics of rice sterile lines. Jiangsu J. Agric. Sci. 41, 625–634. doi: 10.3969/i.issn.1000-4440.2025.04.001

[B20] Li J. Y. Zhou Z. Y. Lan Y. B. Hu L. Zang Y. Liu A. M. . (2015). Distribution of canopy wind field produced by rotor unmanned aerial vehicle pollination operation. Trans. Chin. Soc. Agric. Eng. 31, 77–86. doi: 10.3969/j.issn1002-6819.2015.03.011

[B21] Long L. H. Shu K. (2000). Increasing outcrossing rate of indica hybrid rice. J. Hunan Agric. Univ. (Natural Sciences) 26, 167–170. doi: 10.3321/j.issn:1007-1032.2000.03.004

[B22] Lu L. J. (2023). Study on seed production technology to improve the purity of rapeseed variety Shaanyou28 (Yangling, Shaanxi: Master Dissertation of Northwest A&F University).

[B23] Luo B. L. (2023). Seed industry revitalization and food security: A review. J. South China Agric. Univ. 44, 827–836. doi: 10.7671/j.issn.1001-411X.202309045

[B24] Namai H. Kato H. (1987). The number of pollen grains deposited upon a pistil assuring seed setting of male sterile seed parent in rice (*Oryza sativa* L.). Breed. Sci. 37, 98–102. doi: 10.1270/jsbbs1951.37.98

[B25] Shi W. Zhu G. Y. Sun M. F. Wang A. M. Chen Z. B. Yan G. H. (2020). Influence factors and mechanism of rice grain filling: research progress. Chin. Agric. Sci. Bull. 36, 1–7. doi: 10.11924/j.issn.1000-6850.casb19020022

[B26] Sun Y. B. Chen D. Y. Sun M. F. Yan G. H. Zhu G. Y. Tang H. S. . (2020). Effects of different female parents and transplanting densities on yield of seed production of two-line hybrid rice. Hybrid Rice 35, 24–27. doi: 10.16267/j.cnki.1005-3956.20190710.172

[B27] Tang W. B. Zhang G. L. Deng H. B. (2020). Technology exploration and practice of hybrid rice mechanized seed production. Chin. J. Rice Sci. 34, 95–103. doi: 10.16819/j.1001-7216.2020.9130

[B28] Tao Y. F. Feng S. Q. Jing S. Z. Qin Q. Lei X. L. Wen B. . (2022). Cultivation techniques for mechanized seed production of hybrid rice in Sichuan. Hybrid Rice 37 (1) 98-100, 134. doi: 10.16267/j.cnki.1005-3956.20210120.024

[B29] Wang X. Tang Q. Mo W. (2020). Seed filling determines seed vigour of superior and inferior spikelets during hybrid rice (*Oryza sativa*) seed production. Seed Sci. Technol. 48, 143–152. doi: 10.15258/sst.2020.48.2.01

[B30] Wang R. Y. Wang X. J. Gao L. Yue X. (2022). Trajectory planning of multiple unmanned aerial vehicles for pollination. Chin. Agric. Sci. Bull. 38, 159–164. doi: 10.11924/j.issn.1000-6850.casb2021-0824

[B31] Wei H. H. Zhang X. Zhu W. Geng X. Y. Ma W. Y. Zuo B. Y. . (2024). Effects of salinity stress on grain-filling characteristies and yield of rice. Acta Agronomica Sin. 50, 734–746. doi: 10.3724/SP.J.1006.2024.32021

[B32] Weng X. X. Xu J. D. Wang J. J. Wang G. Huang Y. Zhao J. . (2023). Research on pollination field characteristics of multi-rotor agricultural unmanned aerial vehicle in indica-japonica hybrid rice seed production. Chin. High Technol. Lett. 33, 314–321. doi: 10.3772/j.issn.1002-0470.2023.03.010

[B33] Wu C. Cui K. Hu Q. Wang W. Nie L. Huang J. . (2019). Enclosed stigma contributes to higher spikelet fertility for rice (*Oryza sativa* L.) subjected to heat stress. Crop J. 7, 335–349. doi: 10.1016/j.cj.2018.11.011

[B34] Xiao H. X. Li Y. F. Yuan L. Y. Zhang Z. F. (2021). Application and prospect of China agricultural unmanned aerial vehicle in rice production. Guangdong Agric. Sci. 48, 139–147. doi: 10.16768/j.issn.1004-874X.2021.08.017

[B35] Xu Y. R. Li X. L. Zheng R. L. Deng A. F. Zhu J. J. Yang B. . (2007). Study on different row rations of parents in relationship with yield in seed production of heavy panicle hybrid rice. Seed 27, 103–105. doi: 10.16590/j.cnki.1001-4705.2007.02.070

[B36] Xu X. Shi X. You X. Hao Z. Wang R. Wang M. . (2024). A pair of E3 ubiquitin ligases control immunity and flowering by targeting different ELF3 proteins in rice. Dev. Cell 59, 2731–2744. doi: 10.1016/j.devcel.2024.06.013, PMID: 39025063

[B37] Xu C. C. Wen J. Q. Ji L. Chen Z. D. Fang F. P. (2022). Current Situations, problems and prospects of rice seed industry in China. China Rice 28, 74–78. doi: 10.3969/.issn.1006-8082.2022.05.012

[B38] Xu F. X. Zhang L. Xiong H. Zhu Y. C. Liu M. Jiang P. . (2015). Effects of high temperature during flowering period on seed setting rate and its relationship with sink to source ratios and flowering habit of mid-season hybrid rice. Acta Agronomica Sin. 41, 946–955. doi: 10.3724/SP.J.1006.2015.00946

[B39] Yang J. C. (2010). Mechanism and regulation in the filling of inferior spikelets of rice. Acta Agronomica Sin. 36, 2011–2019. doi: 10.3724/SP.J.1006.2010.02011

[B40] Yi H. Shi H. Mao W. Yin J. Ma Y. Xu L. . (2024). E3 ubiquitin ligase IPI1 controls rice immunity and flowering via both E3 ligase-dependent and -independent pathways. Dev. Cell 59, 2719–2730. doi: 10.1016/j.devcel.2024.06.014, PMID: 39025062

[B41] Yi Z. H. Wu S. G. Xiao C. L. Liu A. M. Chen Z. (2009). Research advance on seed-production characters of male parent of hybrid rice. Hunan Agric. Sci. 39 (7) 17-19, 21. doi: 10.3969/j.issn.1006-060X.2009.07.006

[B42] Zhang C. P. (2022). Effects of planting density on yield and related factors of soybean sterile line (Tongliao, Inner Mongolia: Master Dissertation of Inner Mongolia Minzu University).

