# Supplementary material for: Optimization of row-ratios in mechanized hybrid rice seed production: a study on pollen dispersal and seed-setting characteristics at two ecological sites
Source: Front Plant Sci. 2025 Dec 16;16:1704773. doi: 10.3389/fpls.2025.1704773 (PMC12750148; doi:10.3389/fpls.2025.1704773)
Supplement: Supplementary file 1 [file Supplementaryfile1.docx]

**Table S1** Soil properties of the top soil layer at the ecological sites in Qionglai and Mianzhu

| **Ecological site** | **PH** | **Organic**  **matter**  **(g kg^-1^)** | **Total**  **N**  **(g kg^-1^)** | **Total**  **P**  **(mg kg^-1^)** | **Total**  **K**  **(g kg^-1^)** | **Available**  **N**  **(mg kg^-1^)** | **Available**  **P**  **(mg kg^-1^)** | **Available**  **K**  **(mg kg^-1^)** |
| --- | --- | --- | --- | --- | --- | --- | --- | --- |
| Qionglai | 7.78 | 44.26 | 1.83 | 0.32 | 13.16 | 151.70 | 10.19 | 47.90 |
| Mianzhu | 6.38 | 37.76 | 1.61 | 0.34 | 18.79 | 182.40 | 4.82 | 62.50 |

N, P, and K represent nitrogen, phosphorus and potassium, respectively.

**
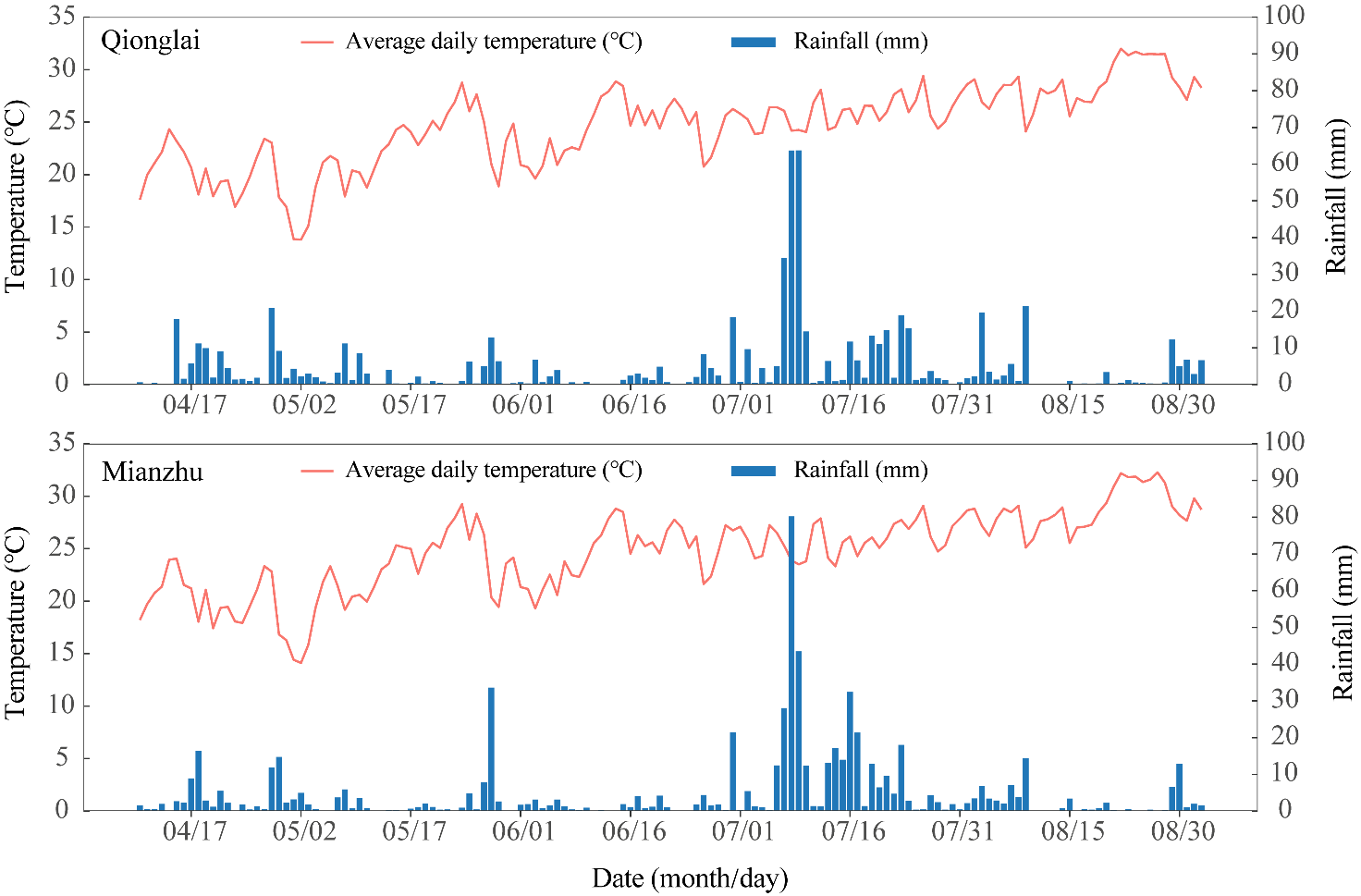
**

**Figure S1** Climate data during the experimental periods in Qionglai and Mianzhu

**Table S2** Parameters of unmanned aerial vehicles

| **Type** | **Weight**  **(kg)** | **Maximum level speed (m s^-1^)** | **Rotor diameter (m)** | **Wheelbase (m)** | **Number of rotor** |
| --- | --- | --- | --- | --- | --- |
| DJI T30 | 36.50 | 10 | 0.97 | 2.15 | 6 |
